# Large-scale combining signals from both biomedical literature and the FDA Adverse Event Reporting System (FAERS) to improve post-marketing drug safety signal detection

**DOI:** 10.1186/1471-2105-15-17

**Published:** 2014-01-15

**Authors:** Rong Xu, QuanQiu Wang

**Affiliations:** 1 Medical Informatics Division, Case Western Reserve, Cleveland, Ohio, USA; 2 ThinTek LLC, Palo Alto, California, USA

## Abstract

**Background:**

Independent data sources can be used to augment post-marketing drug safety signal detection. The vast amount of publicly available biomedical literature contains rich side effect information for drugs at all clinical stages. In this study, we present a large-scale signal boosting approach that combines over 4 million records in the US Food and Drug Administration (FDA) Adverse Event Reporting System (FAERS) and over 21 million biomedical articles.

**Results:**

The datasets are comprised of 4,285,097 records from FAERS and 21,354,075 MEDLINE articles. We first extracted all drug-side effect (SE) pairs from FAERS. Our study implemented a total of seven signal ranking algorithms. We then compared these different ranking algorithms before and after they were boosted with signals from MEDLINE sentences or abstracts. Finally, we manually curated all drug-cardiovascular (CV) pairs that appeared in both data sources and investigated whether our approach can detect many true signals that have not been included in FDA drug labels. We extracted a total of 2,787,797 drug-SE pairs from FAERS with a low initial precision of 0.025. The ranking algorithm combined signals from both FAERS and MEDLINE, significantly improving the precision from 0.025 to 0.371 for top-ranked pairs, representing a 13.8 fold elevation in precision. We showed by manual curation that drug-SE pairs that appeared in both data sources were highly enriched with true signals, many of which have not yet been included in FDA drug labels.

**Conclusions:**

We have developed an efficient and effective drug safety signal ranking and strengthening approach We demonstrate that large-scale combining information from FAERS and biomedical literature can significantly contribute to drug safety surveillance.

## Introduction

Post-marketing drug safety signal detection from spontaneous reporting systems is challenging, demands new types of data, and calls for new avenues for advancing the state-of-the-art in data mining approaches. Mining drug-side effect (drug-SE) associations from the prominent spontaneous reporting system, the US Food and Drug Administration (FDA) Adverse Event Reporting System (FAERS), is a highly active research area. Statistical data mining algorithms such as disproportionality analysis, correlation analysis, and multivariate regression have been developed to detect adverse drug signals from FAERS [1-4]. Currently, domain-specific signal prioritizing and filtering approaches have recently been developed in detecting post-marketing cardiovascular events associated with targeted cancer drugs from FAERS [5]. However, current signal detection methods often suffer from a range of limitations including biased reporting and misattribution of causality in drug-SE combinations [6]. Therefore, it is important to develop robust signal detection methods to identify drug-related adverse events from FAERS. Studies show that complementary data sources such as patient health record (EHR) data can be leveraged upon to improve signal detection from FAERS [4]. In this study, we used over 21 million published biomedical articles to systematically improve signal detection from FAERS. Our study is based on the key assumption that if a drug and a SE co-occur in both FAERS and MEDLINE, it is likely that a true semantic relationship exists between them. A semantic relationship can be, for example, “drug CAUSE SE”, “drug TREAT disease”, or others. In addition, if the pair appears frequently in FAERS, which is a drug adverse events reporting system, then it is more likely to be a “drug CAUSE SE” pair than other relations. We hypothesized that a systematic approach that combined drug safety signals from both biomedical literature and FAERS could augment the discovery of unknown drug-SE association from FAERS.

The main contributions of our study are as follows: (1) We systematically extracted all drug-SE pairs with presence in both FAERS and MEDLINE and showed that these pairs had significantly higher precisions, therefore could be leveraged upon to facilitate signal detection from FAERS; (2) We implemented and compared a total of seven ranking algorithms. We showed that by combining drug safety signals from both FAERS and biomedical literature, some of these algorithms had significantly improved performance; and (3) We have made publicly available a dataset of 269,040 candidate drug-SE pairs that have supporting evidences in both FAERS and MEDLINE. These pairs are highly enriched with true signals that have not been captured in FDA drug labeling to date. Compared to analyses of other data sources such as EHRs or the web, our study used a large amount of published biomedical literature. This data is of high quality, publicly available, and comprised of high quality results from millions of independent scientific studies. To the best of our knowledge, our study is the first large-scale approach to systematically combine data from FAERS and published biomedical literature to facilitate safety signal detection for all drug adverse events reported in FAERS.

## Background

Post-marketing drug adverse events are a major public health problem, accounting for up to 5% of hospital admissions, 28% of emergency visits, and 5% of hospital deaths [7,8], with associated costs of $75 billion annually [9]. Therefore, timely and accurate detection of drug adverse events in real-world patients is critical in improving patients’ quality of life and reducing healthcare costs. Drug safety surveillance has relied predominantly on spontaneous reporting systems, which are composed of both voluntary and mandatory reporting of suspected drug adverse events from health-care professionals, consumers, and pharmaceutical companies. The US Food and Drug Administration (FDA) Adverse Event Reporting System (FAERS) is one of the most prominent spontaneous reporting systems. Mining drug-side effect (drug-SE) relationships from FAERS is a highly active research area. Harpaz et al. recently reviewed the data mining and machine learning approaches to discovering adverse drug events from FAERS [2]. Data mining algorithms such as disproportionality analysis, correlation analysis, and multivariate regression have been developed to detect adverse drug signals from FAERS [1-4]. Recently, researchers began to use other data sources for mining drug-SE associations. For example, patient EHRs have emerged as a promising resource for post-marketing drug adverse event discovery [10-15]. Health information available on the web and web search log data can also provide valuable information on drug side effects [16,17].

Another important information source of drug-SE associations is the vast amount of published biomedical literature. Currently, more than 22 million biomedical records are publicly available on MEDLINE, making it a rich side effect information source for drugs at all clinical stages, including drugs in pre-marketing clinical trials, post-marketing clinical case reports and clinical trials, and many failed drugs. In fact, drug safety researchers have regularly used biomedical literature to evaluate initial signals detected from FAERS [18]. There are several unique advantages to using published biomedical literature for drug safety signal detection over other data sources. First, the number of articles is large (22 million) and included many clinical trials (732,526) and clinical case reports (1,651,631). Second, unlike patient EHRs, biomedical literature is publicly available (all abstracts and many full text articles). Third, in comparison with data collected from the web, the information contained in published biomedical articles is of higher quality. Fourth, unlike information from both EHRs and the web, MEDLINE articles include adverse events information for drugs at all different clinical stages, including investigational, commercial, and even failed drugs. There have been research efforts in mining drug-SE associations from MEDLINE. Shetty et al. applied information mining to discover associations between 35 drugs and 55 SEs from MEDLINE and demonstrated the Vioxx-myocardial infarction associations had been reported in the literature before its withdrawal in 2002 [19]. Gurulingappa et al. trained and tested a supervised machine learning classifier to classify drug-condition pairs in a set of 2972 manually annotated case reports [20]. Both studies focused on a limited set of drugs, side effects or specific article types. It is unclear how these approaches can be scaled up to the whole MEDLINE. In one of our recent studies, we developed an automatic approach to extract anticancer drug-specific side effects from MEDLINE through the development of specific filtering and ranking schemes and demonstrated that the corpus of published biomedical literature contains rich side effect information for cancer drugs [21].

Recently, Harpaz et al. proposed a signal-detection strategy that combined FAERS and EHRs in order to improve the accuracy of signal detection by requiring signaling appeared in both sources [4]. The researchers showed that the approach of combining two large, independent, complementary data sources generated a highly selective ranked set of candidate signals and improved accuracy of signal detection. The researchers used well-established statistical mining approaches to first generate signals from each source. The study focused on signals corresponding to only three adverse reactions (rhabdomyolysis, acute pancreatitis, and QT prolongation).

## Approach

In this study, we systematically combined over 21 million biomedical articles with over 4 million records from FAERS to improve signal detection from FAERS. Our approach was based on the following observations: (1) Drug-SE (or disease) pairs appearing in MEDLINE often have some true semantic relationships such as “drug CAUSE SE”, or “drug TREAT disease” and others. The key issue in extracting drug-SE pairs from literature is to differentiate “drug CAUSE SE” pairs from “drug TREAT disease” pairs, which are dominant in the literature; (2) The majority of the millions of drug-SE associations in FAERS don’t have direct semantic relationship. The key in detecting true signals from FAERS is to differentiate “drug CAUSE SE” pairs from spurious co-occurrence pairs; (3) If a drug-SE pair appears in both MEDLINE and FAERS database, then this pair likely has a true semantic relationship (as determined by its MEDLINE presence). In addition, if this pair also appears in FAERS many times, then the probability of it being a true “drug CAUSE SE” pair is high. Hence, in this study, we implemented a total of seven signal detection approaches, including five currently the most widely used approaches for automated signal detection in FAERS. We also applied the state-of-art adaptive data-driven approach that controlled confounding factors inherent in spontaneous reporting systems [22]. We systematically boosted drug-SE pairs’ original signals in FAERS (as determined by the seven signal detection approaches) by incorporating the information about their MEDLINE presences. Compared to previous studies focused on specific sets of drugs or side effects, our task of processing more than 4 million records from FAERS and 21 million biomedical articles from MEDLINE for millions of drug-SE associations of all drugs and all side effects was more challenging in terms of achieving efficiency, effectiveness, and generalizability.

## Data and methods

**Figure 1:**
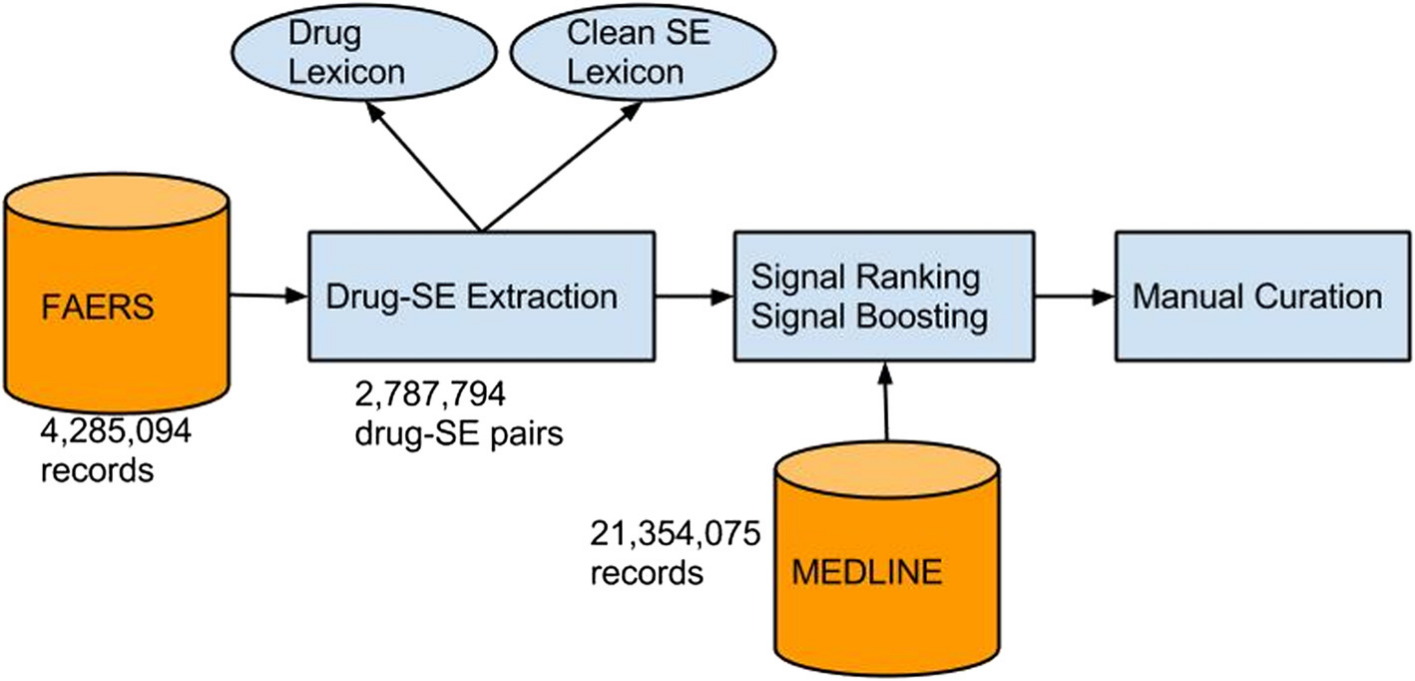
**Data and experimental flowchart.** The two large data sources for drug-SE extraction are 4,285,094 records from FAERS and 21,354,075 MEDLINE records. The process included: (1) drug-SE pair extraction from FAERS; (2) Ranking extracted pairs using six commonly used statistical signal detection approaches, and boosting the rankings by pairs’ MEDLINE presence; and (3) manual curation of all targeted anticancer drug associated cardiovascular events that appeared in both FAERS and MEDLINE and compared them to those captured in FDA drug labeling.

The datasets and experiment flow chart are depicted in Figure 1. The two large data sources for drug-SE extraction are 4,285,094 records from FAERS and 21,354,075 MEDLINE records. The process included: (1) drug-SE pair extraction from FAERS; (2) Ranking extracted pairs using both frequency and six commonly used statistical signal detection approaches, and boosting the rankings by pairs’ MEDLINE presence; and (3) manual curation of all targeted anticancer drug-associated cardiovascular events that appeared in both FAERS and MEDLINE and compared them to those captured in FDA drug labeling.

### Data

#### FDA Adverse Event Reporting System (FAERS)

A total of 4,285,097 records were downloaded from FAERS for the time period from the years 2004 through 2012 were downloaded [23]. Among the downloaded files, files DRUGyyQq.TXT contained drug information associated with reported adverse event. Files REACyyQq.TXT contained all “Medical Dictionary for Regulatory Activities” (MedDRA) terms coded for adverse events. Files DRUGyyQq.TXT and REACyyQq.TXT were the sources for drug-SE association extraction.

#### MEDLINE data and local MEDLINE search engine

We downloaded a total of 21,354,075 MEDLINE records (119,085,682 sentences) published between 1965 and 2012 from the U.S. National Library of Medicine (http://mbr.nlm.nih.gov/Download/index.shtml). Each sentence was syntactically parsed with Stanford Parser [24] using the Amazon Cloud computing service (a total of 3,500 instance-hours with High-CPU Extra Large Instance were used). We used the publicly available information retrieval library Lucene (http://lucene.apache.org) to create a local MEDLINE search engine with indices created on both sentences, their corresponding parse trees and abstracts.

### Methods

#### Extract drug-SE pairs from FAERS

Both high quality drug lexicon and SE lexicon are the prerequisite for subsequent drug-SE pair extraction from FAERS. We built a comprehensive drug lexicon by pooling drug terms (a total of 294,109) from the Unified Medical Language Systems (UMLS 2011AB version). We manually removed many overly general drug names as well as misclassified drug terms. This drug lexicon has been recently used in our study of extracting drug-disease treatment relationships from MEDLINE [25].

We manually created a clean side effect (SE) lexicon from MedDRA, the terminology used in encoding adverse events in FAERS. Many terms in MedDRA are not SE terms themselves. For instance, the MedDRA lexicon contains thousands of medical procedure or lab test terms such as “abdomen scan” and “allergy test”. These terms by themselves are not SE terms. In addition, the MedDRA lexicon includes overly general terms such as “adverse events” and ambiguous terms such as “adhension”. We manually removed these terms from MedDRA. After manual curation, the final clean SE lexicon consisted of 49,625 terms, a significant 29% reduction from the original 70,177 terms. Drug-SE pairs extracted based on this clean SE lexicon should have significantly improved precisions.

We first extracted drug-SE pairs by linking DRUGyyQq.TXT with REACyyQq.TXT through patient report ID numbers. We then cleaned up the extracted pairs as following: (1) Drug entity recognition and mapping: drug names used in DRUGyyQq.TXT often consisted of drug trade names, generic names, or both. In addition, many drug strings were in free text form. We recognized drug entities (both trade names and generic names) from drug strings through a dictionary-based approach. We then mapped all trade names to their corresponding generic names; (2) SE entity recognition: SE entities were recognized from adverse event strings using the clean SE lexicon. After these two steps, we obtained a total of 2,787,797 drug-SE pairs, representing 2,603 drugs and 13,413 SEs.

#### Extract drug-SE pairs that appeared in both FAERS and in MEDLINE

We used each of the 2,787,797 drug-SE pairs extracted from FAERS as a search query to the local MEDLINE search engine. Sentences, their associated parse trees, and abstracts that contained the pair were retrieved. MEDLINE sentence-level drug-SE pairs are those with both drug and SE terms co-occur in the same sentences. MEDLINE abstract-level drug-SE pairs are those with both drug and SE terms co-occur in the same abstracts. Drug-SE pairs in abstract-level include pairs i sentence-level. Instead of simply retrieving a pair’s co-occurrence count from the search engine, we added the extra restriction that both drug and SE terms must be noun phrases in retrieved parse trees. This additional restriction was to prevent the extraction of incorrect drug-SE pairs from sentences. For example, the drug-SE pair “baclofen-decreased activity” appeared in FAERS 19 times. It also appeared in MEDLINE in the following sentence “Although **baclofen decreased activity** during a 30-min period after dosing...”(PMID 2819919). However, the substring “decreased activity” in this sentence is not an SE term. This work in extracting drug-SE pairs that appeared in both FAERS and MEDLINE was computationally intensive and was done using Amazon Elastic Cloud (Amazon EC2) with 1000 parallel instances.

#### Ranking drug-SE pairs by combining signals from both MEDLINE and FAERS

Based on our hypothesis that if a drug-SE pair appeared in both MEDLINE and FAERS, then this pair may have some true semantic relationship. In addition, if the pair also appeared many times in FAERS, a data source mainly for drug adverse events, then the true semantic relationship was more likely to be “drug CAUSE SE” than others. We implemented several signal ranking algorithms, including ranking by pairs’ frequency counts (FREQ) in FAERS, and five commonly used Disproportionality Analysis (DPA) statistical signal detection approaches: relative reporting ration (RRR), proportional reporting ratio (PRR), reporting odds ratio (ROR), phi coefficient (PhiCorr), and information component (IC). The five DPAs are currently the most widely used approaches for automated signal detection in FAERS [2]. All these DPA methods are based on frequency analysis of 2x2 contingency tables to estimate statistical association between drugs and SEs and it intends to quantify the degree to which a drug-SE pair co-occurs disproportionally in the database. These five DPA methods differ by the statistical adjustments they apply to account for low counts. As shown in the Results section, these five DPA methods performed similarly in our study, but had inferior performance than the FREQ-based approach.

It has been demonstrated that DPA approaches may introduce confounding factors that are causing false positives and false negatives [22]. Recently, Tatonetti et al. constructed a dataset called OffSides in which drug side effect associations have confounders partly excluded. We downloaded OffSides at http://www.pharmgkb.org and obtained a total of 438,801 drug-SE pairs from the database. We then ranked these pairs based on values provided in the dataset.

For drug-SE pairs that appeared in both FAERS and MEDLINE, we boosted their ranking scores to the square of their original signals (FREQ, PRR, RRR, ROR, PhiCorr, IC, and OffSides) from FAERS. For drug-SE pairs that appeared in FAERS only, ranks were determined by their original signals in FAERS.

In order to compare different ranking methods, we used the *11-point interpolated average precision*, which is commonly used to evaluate retrieved ranked lists for search engines [26]. For each ranked list, the interpolated precision was measured at the 11 recall levels of 0.0, 0.1, 0.2,..., 1.0. At each recall level, we calculated the arithmetic mean of the interpolated precision. A composite precision-recall curve showing 11 points was then graphed.

In order to compare these seven ranking approaches in ranking known true signals highly among all drug-SE pairs, we used drug-SE pairs from FDA drug labels as the evaluation dataset. Note this evaluation dataset was not used to calculate the true precisions and recalls, but to compare different ranking approaches in prioritize true signals. We used a total of 100,049 drug-SE pairs from the Side Effect Resource (SIDER) [27], a side effect resource compiled from FDA package inserts using text-mining methods, as gold standard.

#### Manual evaluation using evidence from MEDLINE

To demonstrate that drug-SE pairs appearing in both MEDLINE and FAERS are often highly enriched with true signals and that many of these true signals have not been captured in FDA drug labels, we manually curated a subset of the drug-SE pairs that appeared in both FAERS and in MEDLINE: all cardiovascular events (CVs) associated with targeted anticancer drugs. A list of 45 targeted cancer drugs was obtained from the National Cancer Institute (NCI) (http://www.cancer.gov/cancertopics/factsheet/Therapy/targeted). A list of 1,172 CVs was selected from the clean MedDRA-based SE lexicon by finding all leaf nodes with the ancestor “vascular disorders” or “cardiac disorders”. We filtered drug-SE pairs that appeared in both FAERS and MEDLINE sentences with these two lexicons and obtained a total of 617 drug-CV pairs. We used the local MEDLINE search engine to retrieve all the sentences (3,628 in total) wherein these pairs appeared. We then manually classified these 617 drug-CV pairs into three classes (CAUSE, TREAT, and NONE) using the sentences (and abstracts when necessary) as evidence. Three curators with graduate degrees in biomedical sciences performed the curation. Majority vote was used to decide the final classification of each drug-CV pair. Even though the selection of this subset of drug-SE events had certain limitations (i.e. not totally random), however it included many drugs (45 targeted cancer drugs) and many SE terms (1,712 CV terms). In addition, our approach did not favor towards these drug-CV pairs.

## Results

### Named entity recognition (NER) for SEs and drugs

Name entity recognition (NER) for both SEs and drugs is important for the subsequent drug-SE extraction and rankings. For evaluating SE NER, we randomly selected 100 (distinct) SE strings from FAERS and we created a gold standard dataset by manually curated these strings. We compared SE NER on these SE strings using two different SE lexicons: original MedDRA-based lexicon and a manually curated MedDRA-based lexicon (the one used in this study). We show that the precision of NER using the original MedDRA-based lexicon is 0.84, and the precision using the clean lexicon is 1.000. Note that the recalls are 1.000 for both NERs since SE terms in FAERS are encoded with MedDRA terminology. Example errors introduced by using the original MedDRA lexicon are: *abdomen scan*, *adoption*, *aldolase*, *colostomy*, *condom*, and *thyroid operation*. This demonstrated that the manually cleaned SE lexicon significantly contributed to the overall precisions of NER and the subsequent drug-SE pair extraction.

The target of NER is to map drug entities specified in FAERS drug strings (i.e. “erbitux 100 mg imclone /bms”) to their corresponding generic names specified in UMLS (i.e. “cetuximab”). For evaluating drug NER (including both drug name recognition and mapping drug trade names to their generic names), we randomly selected 100 drug strings and manually curated these strings using both UMLS and the web for evidence. We then performed NER on these strings and evaluated the performance. For these 100 drug strings, we correctly mapped 95 of them, and obtained an accuracy of 0.95. The five missed ones are: *thiovalone*, *zoraxin*, *dianeal*, *idroplurivit*, and *UK-427857*. Among the five missed ones, four are not included in UMLS (*thiovalone*, *zoraxin*, *dianeal*, *idroplurivit*). The other one (*UK-427857*) is defined in UMLS, but not included in our drug lexicon since it has the semantic type of “Organic Chemical”. We did not include terms with the semantic type “Organic Chemical” in our drug lexicon because many organic chemicals are not clinical drugs. A total of 39 out of the 100 strings contain no drug entities, majority of which are due to spelling errors. Misspelling examples include: *wrfarin (warfarin)*, *fluorouracl (fluorouracil)*, *ditiazem (diltiazem)*, *cozaril (clozaril)*, *cardine (cardene)*, and *glucosamin (glucosamine)*. Our NER did not try to recognize drug entities from misspelled drug strings. Many of these drug strings that contain spelling errors occur very rarely in FAERS, therefore ignoring them (not trying to identify drug entities from them) will not adversely affect the subsequent signal detection in large degree. The high accuracy of NER for drugs demonstrated that our drug name recognition and mapping approaches are quite effective and contributed significantly to the overall performance of subsequent drug-SE pair extraction from FAERS.

### Drug-SE pairs that appeared in both FAERS and MEDLINE have significantly higher precisions

We extracted a total of 2,787,797 drug-SE pairs from FAERS, among which 125,101 pairs appeared in MEDLINE sentences, and 269,040 pairs appeared in MEDLINE abstracts. We then compared the precisions, recalls, and F1 scores using the known drug-SE pairs from SIDER as the gold standard. Note that this gold standard was not used to measure the actual precisions and recalls. Instead, we use it to demonstrate that pairs appeared in both FAERS and MEDLINE had improved precisions.

As shown in Table 1, drug-SE pairs extracted from FAERS had a recall of 0.507. However, the precision was as low as 0.025. At least two factors may have accounted for this low precision. First, the low precision may be partly caused by false negatives. The gold standard mostly contains drug adverse events reported in controlled clinical trials, therefore could have greatly underestimated the true precision of drug-SE pairs extracted from the post-marketing FAERS. Second, this low precision may have been partly caused by true negatives. The drug-SE pairs were extracted by linking DRUGyyQq. TXT with REACyyQq. TXT through patient report ID numbers. If a patient took m drugs and reported n events, then a total of m x n drug-SE pairs were extracted, many of which may be true negatives.

**Table 1:** Precisions, recalls, and F1 scores of drug-SE pairs that appeared in FAERS alone (“FAERS”), in both FAERS and MEDLINE sentences (“FAERS+sentence”), and in both FAERS and MEDLINE abstracts (“FAERS+abstracts”)

**Source**	**Pairs (n)**	**Precision**	**Recall**	**F1**	
FAERS	2,787,797	0.025	0.507	0.045	
FAERS + sentence	125,101	**0.140**	0.138	**0.139**	
FAERS + abstract	269,040	**0.111**	0.234	**0.151**	

The 125,101 pairs that appeared in both FAERS and MEDLINE sentences had a precision of 0.140, a significant 460% improvement compared to the precision of 0.025 for pairs extracted from FAERS alone. While the recall was lower, the overall F1 score of 0.139 represented a significant 209% improvement. Similarly, the 269,040 pairs that appeared in both FAERS and MEDLINE abstracts had significantly higher precision (0.111 vs. 0.025) and F1 scores (0.151 vs. 0.045). In summary, pairs extracted from FAERS had high recall but low precision. On the other hand, pairs that appeared in both FAERS and MEDLINE had significantly better precisions and F1 scores, but lower recalls. In the sections that follow, we present methods to prioritize true signals from FAERS while at the same time keeping their high recalls. Unlike the previous study by Hapaz, we did not filter out drug-SE pairs that only appeared in FAERS, which may have filtered out many true positives. Instead, we kept all drug-SE pairs while boosting the signals of those pairs that appeared in both data sources.

### Ranking using signals from both FAERS and MEDLINE has better performance in prioritizing true signals

**Figure 2:**
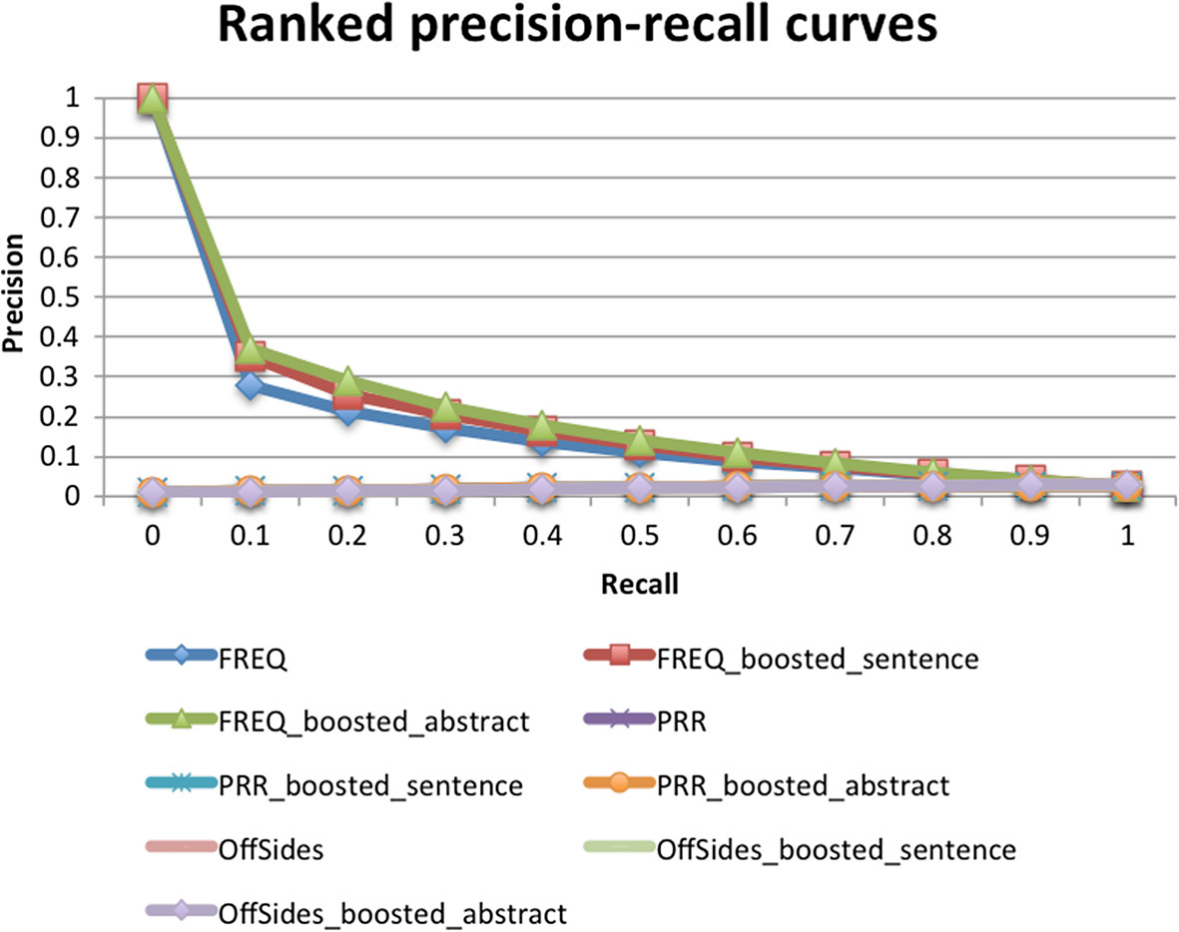
**Precision-recall curves of ranked drug-SE pairs.** The ranked precision-recall curves for pairs ranked by FAERS signals (“FREQ”, “PRR”, “OffSides”) alone, and ranked by FAERS signals augmented by pairs’ presence in MEDLINE (“FREQ_boosted_sentence”, “FREQ_boosted_abstract”, “PRR_boosted_sentence”, “PRR_boosted_abstract”, “OffSides_boosted_sentence”, “OffSides_boosted_abstract”). Rankings by RRR, ROR, IC and PhiCorr had similar performance as that of ranking by PRR (data not shown).

We ranked the 2,787,797 drug-SE pairs extracted from FAERS as follows: if a pair only appeared in FAERS, its rank was its original signal in the FAERS database; if a pair appeared in both FAERS and MEDLINE, its signals was the square of its original signal in FAERS. The ranked precision-recall curves for pairs ranked by FAERS signals (“FREQ”, “PRR”, “OffSides”) alone, and by FAERS signals augmented by pairs’ presence in MEDLINE (“FREQ_boosted_sentence”, “FREQ_boosted_abstract”, “PRR_boosted_sentence”, “PRR_boosted_abstract”, “OffSides_boosted_sentence”, “OffSides_boosted_abstract”) “OffSides_boosted_abstract”) are shown in Figure 2. Rankings by RRR, ROR, IC and PhiCorr had similar performance as that of ranking by PRR (data not shown).

As shown in Figure 2, ranking by frequency (“FREQ”) was effective in ranking known drug-SE pairs highly among those on the list. The precision of top-ranked pairs (at recall of 0.1) by frequency was 0.278, representing a 1,012% increase compared to the precision of 0.025 for all pairs. Ranking by all other six methods had no effect on ranking known drug-SE pairs highly. In fact, many known drug-SE pairs from FDA drug labels are not significant based on PRR or OffSides database. For example, the drug-SE pair “rofecoxib-myocardial infarction” appeared in FAERS a total of 17,306 times. Based on this co-occurrence frequency number only, we are quite certain that it is a true side effect association. However, the same drug-SE pair “rofecoxib-myocardial infarction” is not significant in the OffSides database, even though the more specific pairs “rofecoxib-age indeterminate myocardial infarction”, “rofecoxib-acute myocardial infarction”, and “rofecoxib-silent myocardial infarction” are significant in OffSides.

By leveraging on the signal of a pair’s MEDLINE presence to augment its frequency signal from FAERS, the precisions of drug-SE pairs from FAERS were further improved upon at most of the recalls. For example, when frequency counts of drug-SE pairs were strengthened by their MEDLINE abstract presence (“FREQ_boosted_abstract”), the precision at a recall of 0.1 was 0.371, representing a 33.4% increase as compared to the precision of 0.278 for pairs ranked by frequency alone (“FREQ”). The precision-recall curve for pairs with boosted rankings from MEDLINE sentences has similar results. Note that only 9.6% of pairs (269,040 out of 2,787,797) from FAERS have ever appeared in MEDLINE abstracts and 4.5% of pairs from FAERS have appeared in MEDLINE sentences, therefore we could only boost the signals of at most 9.6% of all FAERS pairs with their MEDLINE presence. Nonetheless, we significantly improved the precision of the top-ranked pairs by 33.4%. Boosting pairs’ ranking signals of PRR or OffSides by their MEDLINE presence had no effect in prioritizing true signals. In summary, ranking by combining pairs’ frequency signals from FAERS and their MEDLINE presence significantly increased the precision of top-ranked pairs.

One of the main sources of false positives is the inclusion of known drug-disease treatment pairs. If a drug-disease treatment pair was included in FAERS, this pair will likely appear in the literature, which is a main source of drug-disease treatment semantic relationships. For example, the drug-disease treatment pair “irinotecan-colorectal cancer” co-occurred in FAERS for 151 times. This pair is highly significant based on all 5 DPA methods as well as the OffSides database (rr= 2.75000000015865, p value < 8.67518006759968e-22). Since this pair also appears in the literature, its original signal will be further boosted. In future studies, we plan to filter out known drug-disease treatment pairs from FAERS database before boosting. This will depend on the availability of a comprehensive and accurate drug-disease treatment relationship database.

### Literature boosting versus EHR boosting

Our study is different from Harpaz’s study [4] as following: (1) while Harpaz’s study used one DPA approach, we implemented a total of six signal ranking algorithms, including ranking by pairs’ frequency counts (FREQ), and five commonly used DPA statistical signal detection approaches. We also used the OffSides database that consists of significant drug-SE pairs with confounders partly excluded. We then compared these approaches before and after being boosted with signals from MEDLINE sentences or abstracts; (2) compared to Hapaz’s study that evaluated three side effects: pancreatitis, rhabdomyolysis, and long QT syndrome, we systematically evaluated our approaches using all drug-SE pairs derived from FDA drug labels; and (3) while Hapaz’s study used evidence from EHR to boost signal detection from FAERS, we used evidence from MEDLINE.

In order to show how the knowledge from MEDLINE overlaps with that from EHRs, we performed the following experiment: we obtained a reference standard that consisted of 18 drug-SE pairs listed in one of the tables in Harpaz’s paper. Among the 18 pairs, however, we can find only 16 of them in FAERS database. For the two missed drug-SE pairs, we found no evidence of associations from original FAERS records. For example, in order to validate *mesoridazine-long QT syndrome* pair that was included in the reference standard, we obtained all original FAERS records that contain substring “mesoridazine” (no NERs for drugs and SEs) and found only the following pairs with frequency counts in FAERS: *mesoridazine (mesoridazine)|mental disorder|1.0*, *mesoridazine besylate|suicide attempt|1.0*, *mesoridazine (mesoridazine)|agitation|1.0*, *mesoridazine (mesoridazine)|tremor|1.0*, and *mesoridazine (mesoridazine)|schizophrenia|1.0*. None of them indicate any association between mesoridazine and long QT syndrome. Similarly, we obtained a total of 1,078 original drug-SE pairs that contain substring “azacitidine”. By manual examination of these pairs, we found no connection between azacitidine and rhabdomyolysis. Therefore, we excluded these two pairs from the reference standard. Of all 16 pairs in the reference standard, 15 pairs co-occurred in MEDLINE sentences, and all 16 co-occurred in MEDLINE abstracts. These results indicate that MEDLINE covered all the pairs in the reference standard, therefore, our approach can boost the signals of all these 16 pairs. However, due to the lack of access to the EHR data, we can not systematically compare the presence of all drug-SE pairs in MEDLINE to that in EHRs. Based on these comparisons, we are still uncertain how addition of EHR data can further boot signal detection in FAERS in the future.

### Many of the drug-CV pairs that appeared in both FAERS and MEDLINE are not included in the FDA drug labels

When evaluated using known pairs derived from FDA drug labels as the gold standard, the drug-SE pairs that appeared in both FAERS and MEDLINE had significantly higher precisions (0.140 vs. 0.025). The question remains as to what the actual precision of these pairs is and how many of them have not been captured in FDA labels.

We systematically curated all 617 targeted cancer drug-CV pairs that appeared in both FAERS and MEDLINE sentences. Targeted cancer drugs are often associated with unexpectedly high cardiovascular toxicity. While FDA drug labels have captured many of these events, spontaneous reporting systems are a main source for post-marketing drug safety surveillance in real-world cancer patients. We retrieved and manually curated all MEDLINE sentences (3,628 in total) where these drug-CV pairs appear. Among the 617 drug-CV pairs that appeared in both FAERS and MEDLINE sentences, 320 pairs were true positive (CAUSE) pairs (precision: 0.519), demonstrating that if a drug-CV pair appears in both FAERS and MEDLINE, it is highly likely to be a true signal. This precision of 0.519 is significantly higher than the precision of 0.140 when known drug-SE pairs from SIDER were used as the gold standard. This demonstrates that using known drug-SE pairs from FDA drug labels could have significantly underestimated the true precision of pairs that appeared in both FAERS and MEDLINE.

More significantly, among the 320 true positive pairs, 258 pairs (80.6%) have not been included in SIDER, demonstrating that many true drug adverse events many have not yet included in FDA drug labels even though there exist copious documentation from both the literature and FAERS. Therefore, focusing on the pairs that appear in both data sources may result in the discovery of many unknown post-marketing drug adverse events.

Among the 617 drug-CV pairs that appeared in both FAERS and MEDLINE, 25.0% are in fact drug-disease treatment pairs (“TREAT”). We examined the “TREAT” pairs and found out that 20% of which are caused by one drug: bevacizumab. Bevacizumab and many other targeted anticancer drugs work by blocking the growth of blood vessels to tumors (angiogenesis). However, these agents also have targets on normal cells, therefore causing many cardiovascular events. Exactly because of their anti-angiogenesis nature, these targeted drugs have been investigated to treat other diseases. For example, bevacizumab has been successfully used to inhibit abnormal VEGF-mediated blood vessel growth around retina in many eye diseases, including as age-related macular degeneration and diabetic retinopathy. In summary, while many targeted cancer drugs cause cardiovascular events in cancer patients, they also are used to treat diseases related to abnormal blood vessel growth. Therefore, these pairs include not only drug-SE causal pairs but also drug-disease treatment pairs. However, we still don’t know if this is true for other types of drugs or side effects.

Among 617 drug-CV pairs, 23.1% have no obvious direct semantic relationships (“drug NONE CV”). Our speculation is that these cardiovascular events may be caused by patients’ co-morbidities. Cancer prevalence is higher in older patients than in younger patients. Older patients also have higher prevalence of cardiovascular diseases. Cardiovascular events in the mis-attributed drug-CV pairs may be caused by cancer patients’ underlying co-morbid cardiovascular diseases.

## Discussion

We presented a large-scale, effective approach to improve signal detection from FAERS. We show that by combining signals from both FAERS and MEDLINE, we significantly improved the drug side effect detection from FAERS. Nonetheless, our study can be improved in several ways. First, even though we used over 21 million MEDLINE records, only about 9.6% of the pairs extracted from FAERS have ever appeared in MEDLINE. Therefore, we could only boost the signals of a small portion of all FAERS pairs with their MEDLINE presence. In addition, we could have further improved the performance if the full-text articles are available and used. Second, corroborative evidence from other data sources such as EHRs and the web data, when combined with the corpus of published biomedical literature, can be used to increase the power of signal detection from FAERS. Our approach is generalizable and can be easily re-targeted to multiple data sources. Third, we showed that the precision of drug-CV pairs for the 45 targeted cancer drugs that have appeared in both FAERS and MEDLINE is as high as 0.519. In addition, more than 80% of them have not been included in SIDER. However, the precisions for other drugs or events may have different precisions and coverage in FDA drug labels. For example, the coverage of adverse events in FDA drug labels for commonly used drugs or drugs in market for a long time may be higher than targeted cancer drugs, many of which were brought to market only in the last ten years. Due to the intense manual curation effort, we were unable to systematically examine all drug-SE pairs that appeared in both FAERS and MEDLINE.

## Conclusions

We presented a large-scale, efficient, and effective approach to improve signal detection from FAERS. Compared to drug side effect detection using signals from FAERS alone, our approach by combining signals from both FAERS and MEDLINE significantly improved the performance. We showed by manual curation that the precisions of drug-SE pairs that appeared in both data sources are highly enriched with true signals. In addition, the majority of these true signals may have not yet been captured in FDA drug labels, even though the supporting evidence is documented in both MEDLINE and FAERS. Our approach is efficient in processing over 4 million records in FAERS and over 21 million articles on MEDLINE. It is effective in ranking true signals highly. Our approach is generalizable and can easily incorporate other text sources such as patient electronic health records (EHRs) or health-related web pages. We have made a list of 179,458 candidate drug-SE pairs (with supporting evidences from both FAERS and MEDLINE) publicly available.

## Data availability

http://nlp.case.edu/public/data/FAERS\_MEDLINE

## Competing interests

The authors declare that they have no competing interests.

## Authors’ contributions

Xu and Wang have jointly conceived the idea, designed and implemented the algorithms, and prepared the manuscript. Both authors read and approved the final manuscript.
